# miR-141 Contributes to Fetal Growth Restriction by Regulating PLAG1 Expression

**DOI:** 10.1371/journal.pone.0058737

**Published:** 2013-03-15

**Authors:** Qiuqin Tang, Wei Wu, Xia Xu, Lu Huang, Qiong Gao, Huijuan Chen, Hong Sun, Yankai Xia, Jiahao Sha, Xinru Wang, Daozhen Chen, Qian Xu

**Affiliations:** 1 Department of Obstetrics, Wuxi Hospital for Maternal and Child Health Care, Wuxi, China; 2 First Clinical Medical College of Nanjing Medical University, Nanjing Medical University, Nanjing, China; 3 State Key Laboratory of Reproductive Medicine, Institute of Toxicology, Nanjing Medical University, Nanjing, China; 4 Key Laboratory of Modern Toxicology of Ministry of Education, School of Public Health, Nanjing Medical University, Nanjing, China; 5 Department of Microbial and Molecular Systems, KULeuven, Leuven, Belgium; 6 Department of Laboratory, Wuxi Hospital for Maternal and Child Health Care, Wuxi, China; Otto-von-Guericke University Magdeburg, Germany

## Abstract

**Background:**

Fetal growth restriction (FGR) is an important but poorly understood condition of pregnancy, which results in significant fetal, neonatal and long-term morbidity and mortality. Novel research has suggested that altered miRNA expression in the plasma and placenta is associated with adverse pregnancy. We hypothesized that aberrant expression of microRNA-141 (miR-141) in the placenta is associated with FGR. Additionally, expression levels of predicted target genes of miR-141 were also analyzed in placental tissues of FGR and normal controls.

**Methodology/Principal Findings:**

Using quantitative real time PCR, we analyzed the expression level of miR-141 and its target genes in placentas of FGR pregnancies (n = 21) and normal controls (n = 34). Western blot was used to detect the protein expression level of the target genes of miR-141. MiR-141 showed significant up regulation in FGR and significant down regulation of its targets, i.e. E2F transcription factor 3 (E2F3) protein, pleiomorphic adenoma gene 1 (PLAG1) mRNA and protein. Moreover, a positive correlation was found between *PLAG1* and *insulin-like growth factor 2* (*IGF2*) expression levels (Spearman *r* = 0.56, *p*<0.0001). MiR-141 yields an AUC of 0.83 with 88.5% sensitivity and 71.7% specificity for separating FGR from normal controls. This study indicates that miR-141 may be diagnostically important in FGR.

**Conclusions/Significance:**

Our results indicate that aberrant high expression level of miR-141 might play important roles in the pathogenesis of FGR by suppressing E2F3 and PLAG1. We propose that miR-141 may participate in a miR-141-PLAG1-IGF2 network relating to FGR development. These findings may provide new targets via miR-141 in diagnosis and therapy of FGR in the future.

## Introduction

Fetal growth restriction (FGR), also known as intrauterine growth restriction (IUGR), is defined as the inability of a fetus to maintain expected growth, with estimated fetal weight or actual birth weight below the 10^th^ percentile for gestational age [1]. It is a relatively common, pleiotropic complication of pregnancy, affecting approximately 5–10% of newborns [2], and it is the second primary cause of perinatal mortality. FGR is associated with significantly increased perinatal morbidity and mortality, and is also a major determinant of cardiovascular disease and glucose intolerance in adult life [3]. Several factors have been identified to be involved in pathogenesis of FGR, including infectious [4], chromosomal alterations [5] or genetic syndromes [6], [7], drug abuse or chemical substances [8], abnormalities of the placenta [9], as well as immunological and anatomical factors [10]. In most cases, however, incomplete placentopoiesis (placental formation) is the cause for the insufficient supply of nutrients and oxygen to the fetus that subsequently causes the deceleration of its growth.

The placenta is of critical importance to ensure the proper growth and development of the fetus while in utero. It is involved in providing the fetus with nutrients and also is involved in waste and gas exchange. The molecular machinery underlying placental development and function is complex, and comprises numerous interconnected regulatory pathways. Mutations of proteins involved in these molecular networks derail murine placental morphogenesis and commonly lead to embryonic lethality or abnormal fetal growth [11].

MicroRNAs (miRNA) are small non-coding RNAs that regulate gene expression through mRNA degradation and translational repression [12]. It has been reported that one third of human genes are regulated by miRNAs [13]; each miRNA can interact with many mRNAs, and conversely each mRNA can be regulated by multiple miRNAs. In recent years, miRNAs have been shown to play a fundamental role in a variety of physiological and pathological processes in human and animals. They can participate to control several biological process including cell growth, apoptosis, development, metabolism, stress adaptation, hormone signaling and differentiation [12], [14], [15].

Currently, most of the miRNA-related studies compare cancer cells versus normal cells, but the analysis of miRNAs in the control of physiological processes including pregnancy is just incipient. Aberrant miRNA expression has been linked to discrete pathologic processes [16]. Current reports have demonstrated specific patterns of miRNAs regulating changes in uterine gene expression [17] or miRNAs that are specifically expressed in embryonic stem cells and whose expression is altered during embryonic development [18]. As the placenta plays a pivotal role in governing fetal development, it is not surprising that placenta expresses numerous types of miRNAs. Whereas many of these miRNAs are ubiquitously expressed, certain miRNA species are very unique to the placenta. Recent reports on miRNA expression profiles in placentas from preeclamptic pregnancies versus normal pregnancies suggested the involvement of some miRNAs in the pathogenesis of preeclampsia [19]. However, the function of miRNAs in FGR is poorly understood. We focused on miR-141 that was previously published to correlate with tumor grade, to be implicated in pregnancy. MiR-141, belonging to the miR-200 cluster, is found up-regulated in nasopharyngeal and ovarian carcinomas in comparison with normal tissues and correlates with poor prognosis [20], [21]. As biological marker, levels of miR-141 are increased in plasma from pregnant women [22]. Therefore, in our present study, we analyzed the expression level of miR-141 in patients with FGR and investigated its potential molecular mechanisms.

## Materials and Methods

### Sample Collection

This study was approved by the Institutional Ethics Committee of Nanjing Medical University. Written informed consent was obtained from each participant. The placental tissue samples, delivered at 37∼42 weeks, were collected from 21 pregnant women complicated with FGR undergoing cesarean section in the Department of Obstetrics and Gynecology of Wuxi Maternal and Child Health Hospital Affiliated to Nanjing Medical University, from January 2011 through December 2011. Placental tissues from 34 normal pregnancies with gestational age matched groups also undergoing selective cesarean section were collected as controls. All patients had normal platelet counts, normal functioning livers and kidneys, and normal blood pressure. All placental samples were frozen within a half hour after delivery and frozen in liquid nitrogen and stored at –70°C until extraction of RNA and protein.

### RNA Extraction and Quantitative Real-time PCR

TRIzol reagent (Invitrogen Technologies Co, USA) was used to isolate the total RNA, all the procedure were performed under the protocol described by the manufacturer. The expression level of miR-141 was quantified with TaqMan RT-PCR assays (Applied Biosystems). RT-PCR reactions were carried out using the manufacturer’s recommendation. In brief, 1 µg of total RNA was reverse transcribed using the TaqMan MicroRNA Reverse Transcription Kit (Applied Biosystems) with miR-141 specific RT-primer (Applied Biosystems). qPCR was performed triplicate in a volume of 10 µl with 384-well plates on ABI Prism 7900HT (Applied Biosystems) with a standard absolute quantification thermal cycling program and using the SDS 2.3 software to determine the cycle threshold (Ct). Cycling conditions were as follows: incubation at 50°C for 2 min, denaturation at 95°C for 10 min, followed by 40 cycles of denaturation for 15 s at 95°C, annealing and extension for 1 min at 60°C. After the reactions, the Ct data were determined using default threshold settings and the mean Ct was determined from the triplicate PCRs. U6 snRNA (RNU6B; Applied Biosystems) served as an endogenous control. The amount of miR-141 was normalized relative to the amount of RNU6B (△Ct = △Ct_ miR-141_-△Ct_ U6_).

A total of 500 ng of total RNAs from placental tissue samples were used for reverse transcription (Takara, Tokyo, Japan) under 37°C for 15 min and 85°C for 30 s. The PCR reaction was performed using the SYBR Green Master Mix (Taraka) in ABI Prism 7900HT (Applied Biosystems, Foster City, CA) with the gene-specific primers. All primer sequences are shown in Table 1. PCR was performed for 5 seconds at 95°C and for 30 seconds at 60°C for 40 cycles. At the end of the PCR cycles, melting curve analyses were performed to validate the specificity of the expected PCR product. Each assay was performed in three independent technical replicates. The results were normalized for the level of *GAPDH*. Relative expression of target genes were calculated using the equation 2^−△Ct^ in which △Ct = △Ct_ (target gene)_ - △Ct *_GAPDH_*.

**Table 1:** Primers used for reverse transcription and quantitative real-time PCR in the placenta.

mRNA	Primer	Sequence
*E2F3*	Forward	5′-AGAAAGCGGTCATCAGTACCT-3′
	Reverse	3′-TGGACTTCGTAGTGCAGCTCT-3′
*PLAG1*	Forward	5′-ATCACCTCCATACACACGACC-3′
	Reverse	5′-AGCTTGGTATTGTAGTTCTTGCC-3′
*TGFβ2*	Forward	5′-CAGCACACTCGATATGGACCA-3′
	Reverse	5′-CCTCGGGCTCAGGATAGTCT-3′
*CUL3*	Forward	5′-AATGCTTGCCAGATGTTA-3′
	Reverse	5′-TTGGTTCTTCCGTTGATT-3′
*ZEB2*	Forward	5′-CAAGAGGCGCAAACAAGCC-3′
	Reverse	5′-GGTTGGCAATACCGTCATCC-3′
*IGF2*	Forward	5′-TTTGTCCCTCTCCTCCTCCA-3′
	Reverse	5′-CAAGGCTCTCTGCCGAAACT-3′
*GAPDH*	Forward	5′-GGAGCGAGATCCCTCCAAAAT-3′
	Reverse	5′-GGCTGTTGTCATACTTCTCATGG-3′

### Western Blot

Protein extracts were prepared from placental tissues using RIPA buffer containing protease inhibitors (cOmplete, ULTRA, Mini, EDTA-free, EASYpack Roche). After extracting, the protein concentration was detected by BCA method. Equal amount of proteins (100 µg) were separated by 12.3% sodium dodecyl sulfate polyacrylamide gel electrophoresis (SDS-PAGE) and transferred to PVDF membrane. Membrane was blocked using 5% skimmed milk and incubated with respective antibodies. Equal amount of protein was confirmed using GAPDH antibody. The primary and secondary antibodies are listed in Table S1. Integrated density values were then calculated using AlphaImager 3400 (Alpha InnoTech, San Leandro, CA, USA). These values were then normalized to GAPDH level.

### miRNA Target Predictions and Pathway Analysis

Potential target genes of miR-141 were identified using the DIANA LAB (http://diana.cslab.ece.ntua.gr/), PicTar (http://pictar.bio.nyu.edu) and TargetScan human 6.0 (http://www.targetscan.org/vert_60/) search engines. Putative target genes were selected for further validation if they were predicted by at least 2 databases mentioned above. The Database for Annotation, Visualization and Integrated Screening (DAVID) (http://david.abcc.ncifcrf.gov) was applied to provide the functional annotation according to the KEGG database (http://www.genome.jp/kegg/).

### Statistical Analysis

The data were presented as mean ± SD. We used the method of 2^−△Ct^ to analyze the result of RT-PCR in all of our experiments. Statistical analysis of gene expression was performed by Mann-Whitney test. To investigate the correlation between miR-141 expression and three target genes, we calculated Spearman correlations for their expression. All statistical analyses were carried out using Stata (Version 9.2, StataCorp, LP), and *p*≤0.05 was considered to be significant.

## Results

### Characteristics of the Sample Population

Fifty five human placental samples were analyzed for the expression of miR-141 and its target genes. The mean (SD) age of FGR and normal controls were 26.20 (3.49) and 27.91 (4.24), respectively. No significant difference was observed between FGR subjects and controls with regard to the age (*p* = 0.127). The demographics of the sample population are given in Table 2.

**Table 2:** Characteristics of the study population.

Characteristic	Control(n = 34)	FGR(n = 21)	*P* Value
Maternal age (y) (mean ± SD)	27.91±4.24	26.20±3.49	NS*
Gestational age at delivery (wks) (mean ± SD)	39.05±1.80	38.09±1.86	NS*
Birth weight (g) (mean ± SD)	3339.06±457.51	2192.38±344.99	<0.001
Apgar score <7 at 5 min	0	0	NS*

* NS: non-significant difference (*p*>0.05).

### miR-141 Expression in FGR and Normal Placental Tissues

**Figure 1:**
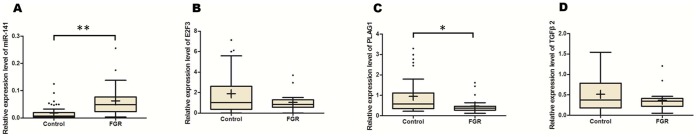
Schematic representation of expression levels of miR-141 and its target genes (*E2F3*, *PLAG1* and *TGFβ2*). MiR-141 and its target genes (*E2F3*, *PLAG1* and *TGFβ2*) were analyzed with real-time reverse transcription polymerase reaction in placentas of FGR pregnancies (n = 21) and normal controls (n = 34). Data are given in Tukey Box plots showing median (−) and mean (+) values. Asterisks denote significant differences from controls (*p<0.05 and **p<0.001).

To determine whether miR-141 expression is associated with FGR, we examined the expression level of miR-141 in FGR placental tissues compared with normal placental tissues using quantitative real-time PCR. The data showed that the expression level of miR-141 in FGR placenta is almost 3.4-fold higher in comparison to the control placenta (Figure 1; *p = *0.0036).

### Expression Levels of miR-141 Target Genes in FGR and Normal Placental Tissues

**Figure 2:**
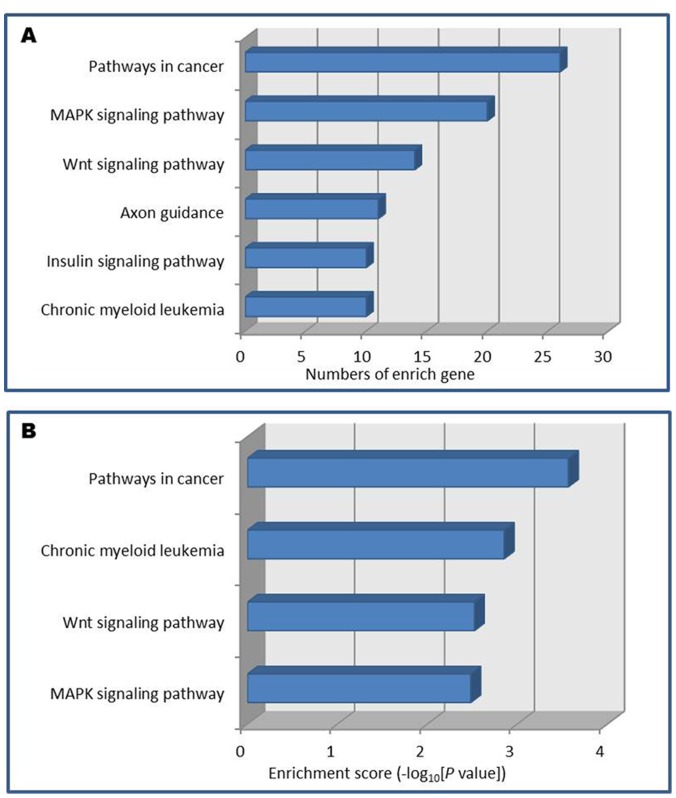
Pathways probably regulated by miR-141. (A) The six pathways were ordered by the number of genes involved in each pathway. (B) Four probably pathways were plotted for genes Enrichment Score >2.0. Enrichment Score reflects in an approximate way the proportion of genes involved in a pathway.

To explore the potential mechanism by which miR-141 executes its function in FGR, we first applied three bioinformatics algorithms (DIANA LAB, PicTar and TargetScan) to identify its potential target genes. Six possible pathways were identified with not less than 10 predicted target genes in each pathway (Figure 2A). As presented in Table S2, four possible pathways were listed with *P* value <0.001 (Figure 2B). They were Pathways in cancer, Chronic myeloid leukemia pathway, Wnt signaling pathway, and MAPK signaling pathway. Finally, we identified five potential targeted genes, i.e. *E2F transcription factor 3* (*E2F3*), *pleiomorphic adenoma gene 1* (*PLAG1*), *transforming growth factor, beta 2* (*TGFβ2*), *cullin 3* (*CUL3*) and *zinc finger E-box binding homeobox 2* (*ZEB2*) for further validation, using at least two of these algorithms. Among the five potential targeted genes, the mRNA expression level of *PLAG1* was significantly decreased in patients with FGR compared with controls (*p* = 0.0076) (Figure 1). However, no significant difference was observed in the expression levels of *E2F3* and *TGFβ2* between FGR patients and normal controls (Figure 1). The expression of *CUL3* and *ZEB2* could not be detected in placental tissue.

### The Relationship between Expression Level of mir-141 and its Target Genes in FGR

**Figure 3:**
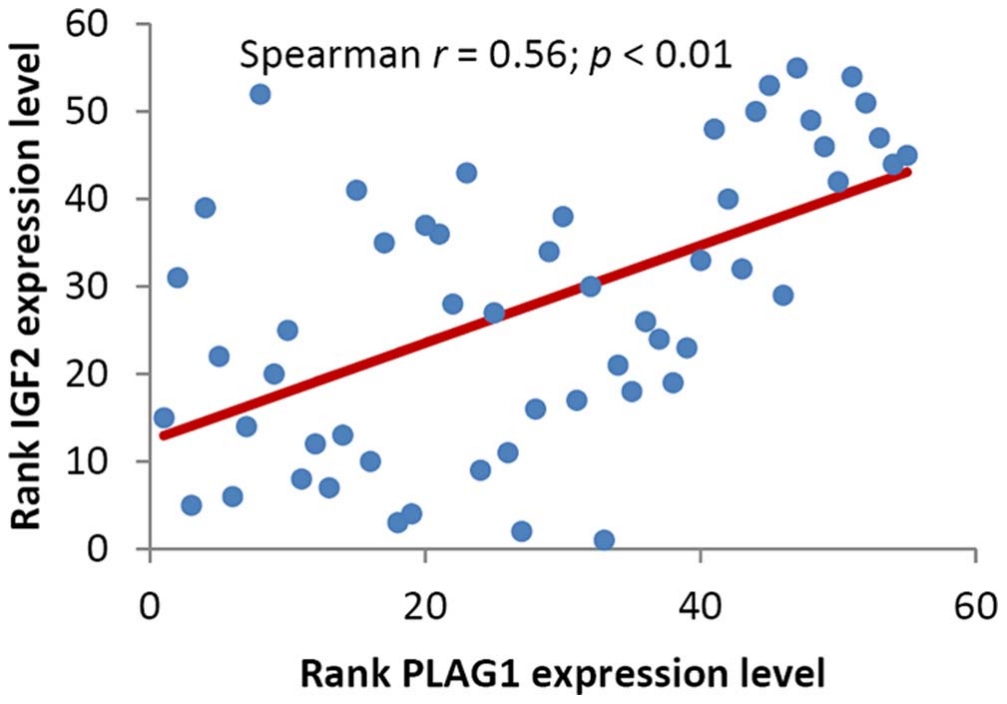
The correlation between expression level of *PLAG1* and *IGF2* expression levels in placentas. The correlation was calculated by Spearman correlation analysis (Spearman *r = *0.56, *p*<0.0001).

As we found that the expression level of *PLAG1* was pronouncedly inhibited in FGR placental tissues compared with normal tissues (Figure 1). We speculated that the reduced *PLAG1* expression in FGR could be a result of the elevated expression level of miR-141. Therefore, spearman correlation analysis was applied to compare the relative expression levels of miR-141 and *PLAG1* in these human placental specimens. There was an inverse correlation between expression of miR-141 and *PLAG1* (Spearman *r* = -0.20; *p* = 0.13), though not reach statistical significance (Figure S1). The up regulation of miR-141 in FGR suppresses the mRNA expression level of *PLAG1*, which may in turns contribute to FGR. Additionally, previous studies have shown that PLAG1 may up regulate the expression of *insulin-like growth factor 2* (*IGF2*), which has been shown to be critical for early human placental development and prenatal growth [23], [24], [25]. We analyses the correlation between expression level of *PLAG1* and *IGF2* in placental tissues of FGR and controls. A statistically significant positive correlation was found between expression of *PLAG1* and *IGF2* (Spearman *r* = 0.56, *p*<0.0001) (Figure 3).

### Protein Expression Levels of miR-141 Target Genes in FGR and Normal Placental Tissues

**Figure 4:**
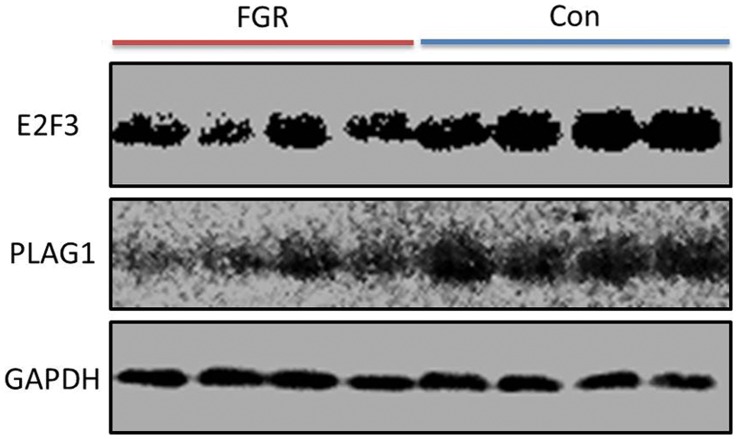
Representation of protein levels of E2F3 and PLAG1 in placentas of FGR pregnancies and normal controls. Expression of E2F3 and PLAG1 protein from placentas were analyzed by Western blots using antibodies in Table 2. Con: normal control.

To further confirm the expression patterns of protein of miR-141 target genes, Western blot was used to measure the relative expression levels. The protein expression levels of E2F3 and PLAG1 in placental tissues were decreased in patients with FGR compared with normal controls (Figure 4). Similar as the mRNA expression levels, no significant change of protein level of TGFβ2 was found between the two groups.

### Receiver Operating Characteristic (ROC) Curves of FGR Prediction with miR-141 Expression

**Figure 5:**
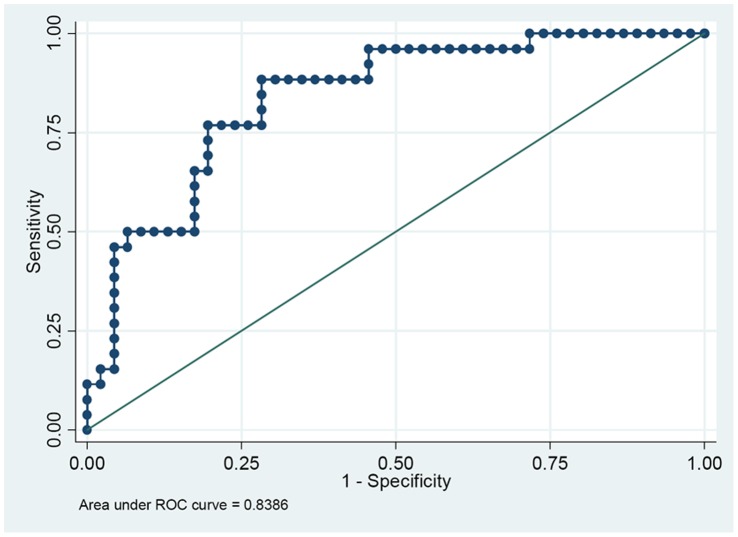
ROC curves for the ability of miR-141 to differentiate the FGR pregnancies (n = 21) from the normal controls (n = 34).

By using the 95% reference interval of miR-141 expression value as risk scores, we constructed the ROC curves and calculated the Area Under the ROC Curve (AUC) to assess the sensitivity and specificity for prediction. As depicted in Figure 5, miR-141 could serve as a potential biomarker to distinguish between FGR and normal controls with AUC of 0.839 and the sensitivity of 88.5% and specificity 71.7% (Figure 5).

## Discussion

Recent reports have quantitatively analyzed the expression of up to 820 miRNAs in placental tissue samples collected in the first or third trimester. Interestingly, the concentration of pregnancy-associated miRNAs increased throughout pregnancy and was altered in placentas from pregnancies with preeclampsia or preterm labor [26], [27]. These results suggest miRNAs as potential serum markers for the normal function of the placenta. However, little is known about the miRNAs expression levels in placental tissue of FGR.

We provide the first evidence that the involvement of miR-141 in the pathology of FGR disease. MiR-141, belonging to the miR-200 cluster, is found to correlate with tumor grade, to be implicated in pregnancy. Chim et al. [28] suggested that several placental miRNAs (miR-141, miR-149, miR-299-5p, and miR-135b) were highly expressed in maternal plasma during pregnancy and noted that such expression patterns may serve as clinical biomarkers for pregnancy monitoring. However, they did not find any significant differences between FGR patients and controls with relative small sample size. In our present study, we demonstrated that FGR patients have higher expression level of miR-141 compared with normal controls. Furthermore, we analyzed five predicted miR-141 target genes expression and found that mRNA expression of *PLAG1* was significantly decreased and the protein expression was also decreased. The protein expression of PLAG1 was consistent with the pattern observed at the mRNA level, indicating that miR-141 repress PLAG1 at both transcriptional and post-transcriptional level. However, the expression of E2F3 was only repressed by miR-141 particularly at the post-transcriptional level.

The E2F transcription factors have emerged as critical apoptotic effectors. The E2F family is essential for extra-embryonic cell proliferation, placental development, and fetal viability [29]. The E2F family member E2F3 protein contributes to control of proliferation in *Rb* mutant embryos in a tissue-specific manner, and plays a major role in the placenta and nervous system [30]. *E2F3* is highly expressed in adult human tissues and is required for the appropriate development of placental tissues [31]. Additionally, the *Plag1* proto-oncogene encodes a transcription factor and is implicated in human tumorigenesis via ectopic overexpression [32]. *PLAG1* is located on human chromosome 8q12 whose oncogenic activation is a crucial event in the formation of various human tumors, including pleomorphic adenomas of the salivary glands [33], lipoblastoma [34], and hepatoblastomas [35]. PLAG1 is expressed mainly during embryonic development and is highly expressed in placenta [36]. Hensen et al. [32] found that targeted disruption of the murine *Plag1* could cause growth retardation. In recent microarray analyses, genes were identified that are consistently induced or repressed by *PLAG1*, and these were classified into various functional categories. Among the classes of up-regulated *PLAG1* targets, the one encoding growth factors was the largest and included the genes for *IGF2*, *cytokine-like factor 1* (*CLF1*), *bone-derived growth factor* (*BPGF1*), *choriogonadotropin beta chain* (*CGB*), *vascular endothelial growth factor* (*VEGF*) and *placental growth factor* (*PGF*) [37]. In the present study, we also found that the expression level of *PLAG1* was strongly positive correlated with the expression level of *IGF2* in placental tissues of FGR and normal controls. These results was also consistence with previous report that the *IGF2* mRNA level was significantly decreased in the placental tissue from the FGR pregnancies [38]. Thus, we propose that miR-141 may participate in a miR-141-PLAG1-IGF2 network relating to FGR development.

Using the KEGG database we searched for pathways where the miR-141 target genes function in order to gain insight into the processes that could be affected by the dysregulated miR-141. The target genes of miR-141 were involved in several important signaling pathways, i.e. MAPK signaling pathway and Wnt signaling pathway. MAPK signaling pathway is implicated in regulating cell growth and differentiation [39]. Decreased MAPK signaling can contribute to defective placental development [40]. Mutation in this signaling cascade lead to defects in the placental labyrinthine region [41]. Additionally, Wnt signaling has been implicated in the regulation of cell proliferation, apoptosis and cell fate [42]. This pathway also played a critical role in both initiating and patterning of the anterior-posterior axis, which precedes gastrulation during mouse embryonic development. Aberrant expression of miR-141 might inactivate these two important pathways, consequently affecting placental development and fetal growth. Further study of MAPK and Wnt pathway may have implications for understanding the pathogenesis of FGR.

Downstream effects of miRNA on post-transcriptional gene regulation remain more challenging and require use of bioinformatics approaches to first predict mRNA targets of specific miRNA and then confirm the effects of over- or under-expression of miRNA on that particular target. Due to the miRNAs’ ultimate role in controlling protein translation, true confirmation of targets requires examination of the proteins of interest using specific antibodies, or through in-vitro approaches coupling targeted miRNA binding regions to reporter constructs. The understanding of miRNAs’ role in the human placenta is in its infancy but due to the known critical role of miRNA in human development, it is certainly an attractive and exciting field of study.

Our study has several limitations. The most important limitation is the size of the population studied. The sample size of our present study was relatively small. Future studies with larger sample size are needed to validate this association. Secondly, our results were based on unadjusted estimates, while a more precise analysis should be conducted if all individual data were available, which would allow for the adjustment by other co-variants including age, body mass index, smoking status, drinking status, and other lifestyle factors. Thirdly, only one miRNAs were involved in our study, while more miRNAs should be evaluated in the future, for instance identifying the miRNAs expression profiling using microarray assay.

Our data are the first to indicate that miR-141 was significantly upregulated in placental tissues of FGR compared with normal controls. In particular, we have demonstrated that the significantly increased miR-141 could serve as adjunct biomarkers for the diagnosis of FGR. Additionally, we propose the effect of up-regulated miR-141 contribute to FGR may through down regulation of *E2F3* expression at post-transcriptional level and PLAG1 expression at both transcriptional and post-transcriptional level. These results provide novel information regarding the molecular mechanism of FGR and the clinical value of miR-141 pertinent to FGR. Further in-depth studies are required to unravel the functional significance of miR-141 in molding placental robustness, which must constantly adapt to altered maternal physiologic status to sustain optimal support to the developing embryo.

## Supporting Information

Figure S1 **The correlation between expression level of** ***E2F3*** **(A),** ***PLAG1*** **(B),** ***TGFβ2*** **(C) and miR-141 expression levels in placentas.** The correlation was calculated by Spearman correlation analysis.
(TIF)

Table S1 **The primary and secondary antibodies of miR-141 target genes and GAPDH were used in Western blot.**
(DOC)

Table S2 **Pathways probably regulated by miR-141 in FGR.**
(DOC)
