# Supplementary figures and images for: miR-141 Contributes to Fetal Growth Restriction by Regulating PLAG1 Expression
Source: PLoS One. 2013 Mar 15;8(3):e58737. doi: 10.1371/journal.pone.0058737 (PMC3598866; doi:10.1371/journal.pone.0058737)

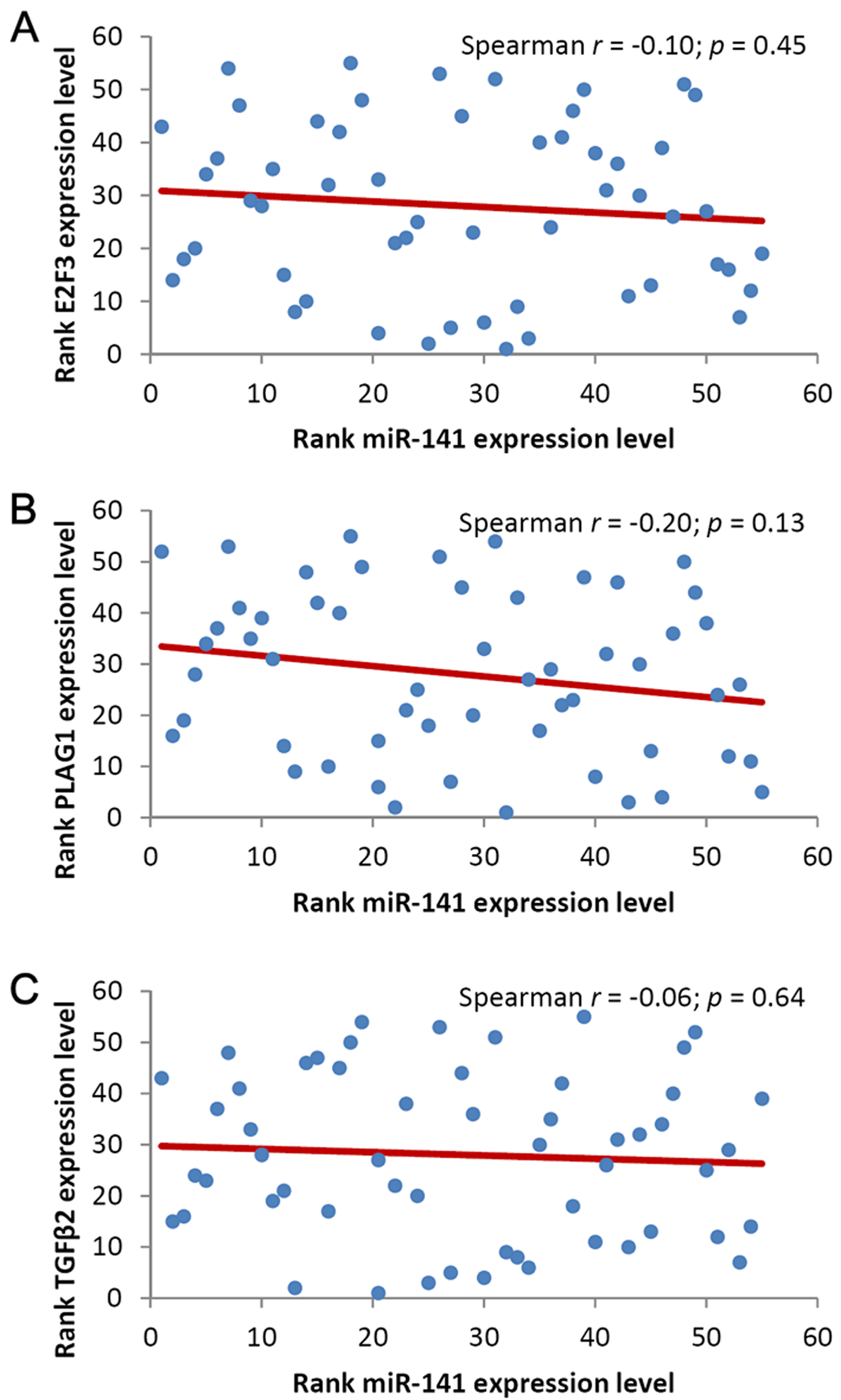

Supplement: Figure S1 — The correlation between expression level of E2F3 (A), PLAG1 (B), TGFβ2 (C) and miR-141 expression levels in placentas. The correlation was calculated by Spearman correlation analysis. (TIF) [file pone.0058737.s001.tif]
