# Supplementary material for: miR-141 Contributes to Fetal Growth Restriction by Regulating PLAG1 Expression
Source: PLoS One. 2013 Mar 15;8(3):e58737. doi: 10.1371/journal.pone.0058737 (PMC3598866; doi:10.1371/journal.pone.0058737)
Supplement: Table S1 — The primary and secondary antibodies of miR-141 target genes and GAPDH were used in Western blot. (DOC) [file pone.0058737.s002.doc]

Table S1. The primary and secondary antibodies of miR-141 target genes and GAPDH were used in Western blot.

| **Protein** | **Primary antibody** | **Dilution ratio** | **Secondary antibody** | **Dilution ration** |
| --- | --- | --- | --- | --- |
| E2F3 | rabbit polyclonal IgG (N-20 sc-879, Santa Cruz Biotechnology, CA, USA) | 1:200 | donkey anti-rabbit IgG-HRP (sc-2313, Santa Cruz Biotechnology, CA, USA) | 1:1000 |
| PLAG1 | goat polyclonal IgG (S-15 sc-20320, Santa Cruz Biotechnology, CA, USA) | 1:100 | donkey anti-goat IgG-HRP (A0181 Beyotime, China) | 1:1000 |
| GAPDH | GAPDH antibody (AG019-1 Beyotime, China) | 1:1000 | goat anti-mouse IgG-HRP (A0216 Beyotime, China) | 1:1000 |
