# Supplementary material for: miR-141 Contributes to Fetal Growth Restriction by Regulating PLAG1 Expression
Source: PLoS One. 2013 Mar 15;8(3):e58737. doi: 10.1371/journal.pone.0058737 (PMC3598866; doi:10.1371/journal.pone.0058737)
Supplement: Table S2 — Pathways probably regulated by miR-141 in FGR. (DOC) [file pone.0058737.s003.doc]

Table S2. Pathways probably regulated by miR-141 in FGR.

| **Term** | **Count** | **P Value** | **Benjamini** |
| --- | --- | --- | --- |
| Pathways in cancer | 26 | 2.7E-4 | 3.4E-2 |
| Chronic myeloid leukemia | 10 | 1.4E-3 | 8.7E-2 |
| Wnt signaling pathway | 14 | 3.03-3 | 1.2E-1 |
| MAPK signaling pathway | 20 | 3.3E-3 | 1.0E-1 |

Note: Probabilities were evaluated by Bonferroni correction and values less than 0.001 were considered significant. Count represents targeted genes involved in the term.
